# The nuclear factor kappa B signaling pathway is a master regulator of renal fibrosis

**DOI:** 10.3389/fphar.2023.1335094

**Published:** 2024-01-16

**Authors:** Na Ren, Wen-Feng Wang, Liang Zou, Yan-Long Zhao, Hua Miao, Ying-Yong Zhao

**Affiliations:** ^1^ The First School of Clinical Medicine, Shaanxi University of Chinese Medicine, Xianyang, Shaanxi, China; ^2^ School of Pharmacy, Heilongjiang University of Chinese Medicine, Harbin, China; ^3^ School of Food and Bioengineering, Chengdu University, Chengdu, Sichuan, China; ^4^ Dialysis Department of Nephrology Hospital, Shaanxi Traditional Chinese Medicine Hospital, Xi’an, Shaanxi, China; ^5^ School of Pharmacy, Zhejiang Chinese Medical University, Hangzhou, Zhejiang, China

**Keywords:** nuclear factor kappa B, renal fibrosis, inflammation, acute kidney injury, chronic kidney disease, natural products

## Abstract

Renal fibrosis is increasingly recognized as a global public health problem. Acute kidney injury (AKI) and chronic kidney disease (CKD) both result in renal fibrosis. Oxidative stress and inflammation play central roles in progressive renal fibrosis. Oxidative stress and inflammation are closely linked and form a vicious cycle in which oxidative stress induces inflammation through various molecular mechanisms. Ample evidence has indicated that a hyperactive nuclear factor kappa B (NF-ƙB) signaling pathway plays a pivotal role in renal fibrosis. Hyperactive NF-ƙB causes the activation and recruitment of immune cells. Inflammation, in turn, triggers oxidative stress through the production of reactive oxygen species and nitrogen species by activating leukocytes and resident cells. These events mediate organ injury through apoptosis, necrosis, and fibrosis. Therefore, developing a strategy to target the NF-ƙB signaling pathway is important for the effective treatment of renal fibrosis. This Review summarizes the effect of the NF-ƙB signaling pathway on renal fibrosis in the context of AKI and CKD (immunoglobulin A nephropathy, membranous nephropathy, diabetic nephropathy, hypertensive nephropathy, and kidney transplantation). Therapies targeting the NF-ƙB signaling pathway, including natural products, are also discussed. In addition, NF-ƙB-dependent non-coding RNAs are involved in renal inflammation and fibrosis and are crucial targets in the development of effective treatments for kidney disease. This Review provides a clear pathophysiological rationale and specific concept-driven therapeutic strategy for the treatment of renal fibrosis by targeting the NF-ƙB signaling pathway.

## 1 Introduction

Renal fibrosis has become a significant public health problem because of its high morbidity and mortality worldwide (Mantovani and Zusi, 2020; Kalantar-Zadeh et al., 2021; Yu et al., 2022). Acute kidney injury (AKI) and chronic kidney disease (CKD) are the most common kidney diseases that can lead to renal fibrosis (hAinmhire and Humphreys, 2017). AKI is an extraordinarily dangerous clinical syndrome due to the rapid decline in kidney function and tubulointerstitial inflammatory cell infiltration, which leads to the accumulation of end products such as creatinine and urea, resulting in a decrease in urine output (Reid and Scholey, 2021). Accumulating evidence indicates that renal tubular cells have an impact on AKI-mediated inflammation, which often results in increased mortality rates, hospitalization time, and medical-related costs (Hoste et al., 2018). Importantly, episodes of AKI are associated with short-term adverse outcomes, and advanced AKI may also result in the development of CKD and even end-stage renal disease (ESRD) (Song et al., 2021). ESRD is a worldwide socioeconomic burden, and patients require renal replacement therapy through dialysis or kidney transplantation (Webster et al., 2017; Choueiri et al., 2022). Moreover, AKI and CKD cause chronic inflammation, which can lead to renal fibrosis (Jin et al., 2021; Nikolic-Paterson et al., 2021). In fact, renal fibrosis is accompanied by the excessive accumulation of extracellular matrix (ECM) proteins, such as fibronectin (FN) and various collagens, in the glomerulus and renal tubulointerstitium (Zhao et al., 2020; Ren et al., 2023b). Regardless of the etiology, renal fibrosis is a chronic and progressive process that leads to a decline in renal function during CKD (Miao et al., 2020; Miao et al., 2022a). Oxidative stress and inflammation play central roles in progressive renal fibrosis. Oxidative stress and inflammation are closely linked and form a vicious cycle in which oxidative stress induces inflammation through various underlying molecular mechanisms (Wang et al., 2023b). Nuclear factor kappa B (NF-ƙB) affects various types of cells and is important for inflammation, the immune response, the cell cycle, and cell survival (Shih et al., 2015). Numerous publications have suggested that the NF-ƙB signaling pathway regulates the inflammatory response and is associated with the pathogenesis of renal fibrosis (Zhang and Sun, 2015; Song et al., 2019). Thus, the potential of NF-ƙB as a drug target for the treatment of renal fibrosis could lead to more specific concept-driven therapeutic strategies. This Review discusses the roles of the NF-ƙB signaling pathway in both AKI and CKD, as well as the possibility of therapeutically targeting the NF-ƙB signaling pathway in renal fibrosis.

## 2 Renal fibrosis

Fibrosis is an enormous burden that affects 25% of the world’s population and can contribute to the failure of organ structural integrity, functional impairment, and even death (Chen T. K. et al., 2019; Rashid et al., 2022). The annualized incidence of major fibrosis-related conditions is nearly 1 in 20 (Zhao et al., 2020). Approximately 45% of total disease-related deaths are associated with abnormal fibroblast activation and fibrosis (Distler et al., 2019). Similarly, renal fibrosis is a final common stage in most CKD cases and results from renal injury when the wound healing and repair processes are dysregulated (Ren et al., 2023a). It involves inflammatory cell infiltration, intrinsic renal cell damage and apoptosis, cell phenotypic transition to fibroblasts and myofibroblasts, abnormal secretion of inflammatory cytokines, and ECM deposition (Yang et al., 2019). Physiologically, the phenotypic transition between epithelial cells and fibroblasts/myofibroblasts is fundamental for tissue development and homeostasis. However, abnormal epithelial-mesenchymal crosstalk results in the formation of a profibrotic milieu, which inhibits the normal wound repair process. Repetitive or persistent injury to the epithelium initiates HYPERLINK “https://www.sciencedirect.com/topics/pharmacology-toxicology-and-pharmaceutical-science/fibrosis” \o “Learn more about fibrosis from ScienceDirect’s AI-generated Topic Pages” fibrosis (Prasad et al., 2014). The kidney can be divided into three main compartments, the tubulointerstitial system, glomerular system and vascular system, each of which may be exposed to fibrosis such as glomerulosclerosis, tubulointerstitial fibrosis, vasculoarterial sclerosis, and perivascular fibrosis (Djudjaj and Boor, 2019). Sclerotic lesions in the glomerular tuft predominantly consist of capillary basement membrane proteins with smaller amounts of fibrillar collagens III and V, whereas periglomerular fibrosis and fibrosis within glomerular crescents typically contain higher levels of collagen I (Duffield, 2014). Interstitial fibrosis is defined as the excessive synthesis and deposition of ECM components and is associated with inflammatory cell infiltration, tubular epithelial cell damage, fibroblast activation and proliferation, and rarefaction of the peritubular microvasculature (Yu et al., 2022). In most cases, renal fibrosis is closely associated with tubular cell injury, which can progress to tubular atrophy, as shown by renal biopsy. At this stage, we consider tubular (and nephron) damage irreversible. In many cases, interstitial fibrosis and tubular atrophy occur in parallel, which leads to interstitial fibrosis (Djudjaj and Boor, 2019). Vasculoarterial sclerosis and perivascular fibrosis are fibrous thickenings of the intima and extima of the vascular network, respectively (Djudjaj and Boor, 2019). Fibrosis involves nearly all types of cells in the kidneys (pericytes, endothelial cells, mesangial cells, and podocytes), as well as macrophages and fibroblasts, and different pathways are involved in the pathogenesis of renal fibrosis, illustrating the immense complexity of this process (Liu, 2011; Feng et al., 2020). In summary, excessive fibrosis is a key factor in the development of kidney disease, and it is an irreversible progression that can impair renal function and cause severe organ failure (Li et al., 2021).

### 2.1 Mechanisms of renal fibrosis

Renal fibrosis is a characteristic final stage of inflammation that occurs in almost all renal diseases (Lin et al., 2020). Fibrosis affects all areas of the kidney, eventually leading to renal parenchymal destruction and ESRD (Gusev et al., 2021; Malaki, 2022). The process of renal fibrosis involves five stages. First, inflammation and massive mononuclear/macrophage infiltration activate the renal tubular epithelium. Macrophages are divided into M0, M1, and M2 types. M1 macrophages promote the Th1-type inflammatory response by secreting inflammatory factors such as interleukin-1 (IL-1), interleukin-6 (IL-6), interleukin-12, and tumor necrosis factor-alpha (TNF-α) (Yang et al., 2021). Second, excessive production of fibrogenic cytokines and growth factors, such as transforming growth factor (TGF)-β and connective tissue growth factor (CTGF), occurs (Nastase et al., 2018). Third, an imbalance in the compounding and degrading of ECM occurs, and excessive ECM accumulates in the renal interstitium, which is the main stage of renal structural and functional damage. Key mediators that drive EMT conversion, such as TGF-β/Smads, interleukins, Wnt/β-catenin, Twist1, and Snail1, are activated in renal tubular epithelial cells after injury (Sun et al., 2016). Fourth, intrinsic renal cell interstitial alterations occur, which are accompanied by a reduction in the number of intrinsic kidney cells. Finally, renal microangiopathy results in renal interstitial ischemia and hypoxia (Wei et al., 2022). Renal fibrosis is characterized by tubular loss and ECM accumulation, and myofibroblasts, which are a potent and effective form of fibroblast, are often regarded as the primary source of ECM production during renal fibrosis (Wu et al., 2022). During fibrosis, aberrant fibroblast activation and the expression of α-SMA indicate that myofibroblasts contribute significantly to the pathogenesis of kidney fibrosis. After injury, inflammatory cells infiltrate renal tissue, and the subsequent production of inflammatory cytokines activates fibroblasts to proliferate and produce extracellular matrix proteins, such as collagen I, III, IV, and FN (Sun et al., 2016). Damaged renal tubules and invading inflammatory cells produce profibrotic factors, resulting in the activation of myofibroblasts through paracrine or autocrine mechanisms (Quan et al., 2020). In addition to immunological factors, a wide array of non-immunologic elements, such as reactive oxygen species (ROS) and advanced glycation end products (AGEs), as well as diseases such as hyperglycemia, hypertension, and hypoxia, influence the development of renal fibrosis (Meng et al., 2014; Wu et al., 2021; Wang et al., 2023c). Oxidative stress is enhanced in CKD patients, especially those with diabetic kidney disease. An imbalance between ROS production and scavenging occurs through dysfunctional mitochondrial respiration (Honda et al., 2019). AGEs are slowly degraded with blood glucose control under hyperglycemic conditions. Furthermore, receptor for advanced glycation end products (RAGE) expression is increased in aging kidneys and DN, and the increase in RAGE expression mediates the sustained activation of oxidative stress and inflammation via the NF-**ƙ**B signaling pathway (Rungratanawanich et al., 2021).

### 2.2 Renal fibrosis and inflammation

**FIGURE 1:**
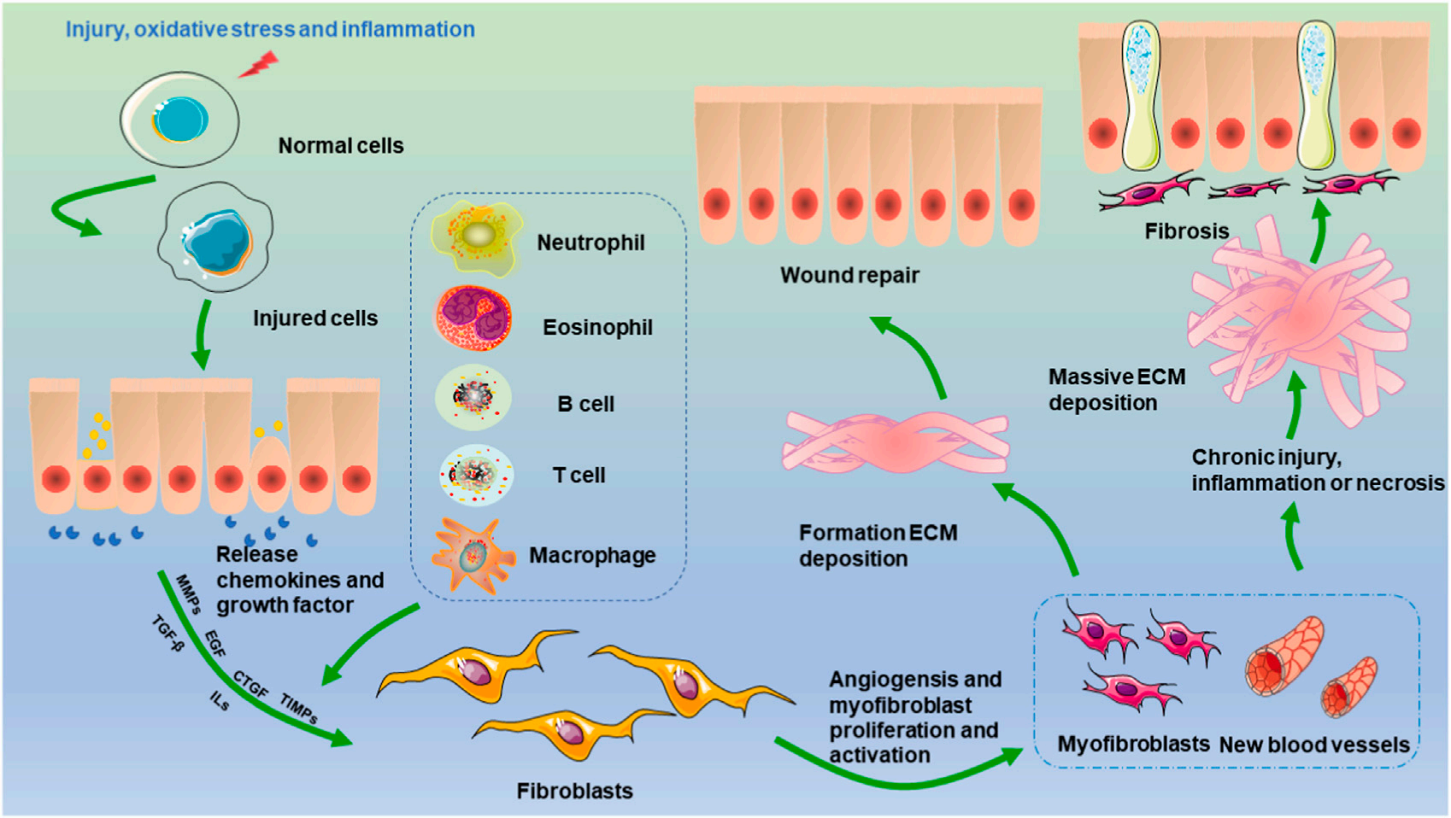
Mechanisms of renal fibrosis. Injury, oxidative stress, and inflammation stimulate the transformation of epithelial cells, endothelial cells, and pericytes. The activation of monocytes and macrophages leads to the production of cytokines and chemokines, which stimulate the activation of fibroblasts. Two events occur once activated fibroblasts are changed into myofibroblasts. One is the healing of damaged tissue, which is followed by wound contraction and regrowth of the epithelium. On the other hand, when persistent damage, an inflammatory response, and necrosis occur, myofibroblasts become permanently active, excessive ECM is produced, and renal fibrosis ultimately occurs.

A wide range of noxious irritants, such as dysmetabolism, inflammation, autoimmune infection, and trauma, may lead to the dysregulation of various molecular pathways that initiate and drive fibrosis (Feng et al., 2019b; Djudjaj and Boor, 2019; Wang et al., 2023c; Rashid et al., 2023). Extensive evidence has suggested that the inflammatory response plays an integral role in the development of renal fibrosis (Chen et al., 2016; Luo et al., 2021) (Figure 1). Acute inflammation, which is the initial period of inflammation, is mediated by the activation of the immune system, is an essential part of the innate defense mechanism, is short in duration, and is typically beneficial to the host (Sobhon, 2023a). In the context of acute inflammation, the initiation of an inflammatory response occurs because of a stimulus, which subsequently leads to the release of various cytokines and chemokines, such as IL-1, IL-6, interferon gamma, and TNF. These molecules play pivotal roles in driving localized and systemic responses. Additionally, immune cells, particularly macrophages and neutrophils, are recruited and proliferate. Ultimately, the threat is eliminated, allowing for the restoration of baseline conditions and subsequent tissue repair (Bennett et al., 2018). However, if acute inflammation worsens and cannot be controlled, a second phase (chronic inflammation) is triggered, which may predispose the host to a number of chronic diseases, including fibrosis (Bennett et al., 2018; Tan et al., 2022; Sobhon, 2023b). Although acute inflammation is beneficial in the early stages of kidney injury, chronic inflammation leads to renal fibrosis. Kidney injury induces an inflammatory response that is protective, and a sustained inflammatory response can contribute to renal fibrosis. During this process, leukocytes and fibrotic cells are recruited to the glomerulus and renal interstitium and activate resident renal immune cells, and this recruitment contributes to increased levels of proinflammatory cytokine production (Chung et al., 2023). The chemokine gradient further drives the infiltration of monocytes/macrophages, T cells, and B cells toward the site of injury. Moreover, chemokines are mediators of angiogenesis, fibroblast recruitment, and epithelial-to-mesenchymal transition (Lv et al., 2018). In addition, a proportion of bone marrow-derived macrophages are transformed into myofibroblasts through macrophage-to-myofibroblast transition (MMT), and these cells coexpress a macrophage marker (CD68) and alpha-smooth muscle actin, produce collagen I, and promote renal fibrosis (Lan, 2022). A recent study suggested that bone marrow-derived macrophages could also lead directly to renal fibrosis via MMT. For instance, *in vitro* and *in vivo* experiments have indicated that macrophages infiltrating the glomerulus in patients with diabetic nephropathy can be converted to myofibroblasts through MMT, ultimately leading to renal fibrosis (Wei et al., 2022). Currently, several approaches to protecting against renal fibrosis through an anti-inflammatory pathway have recently attracted increased attention (Stenvinkel et al., 2021).

## 3 NF-ƙB signaling pathway-mediated inflammation

**FIGURE 2:**
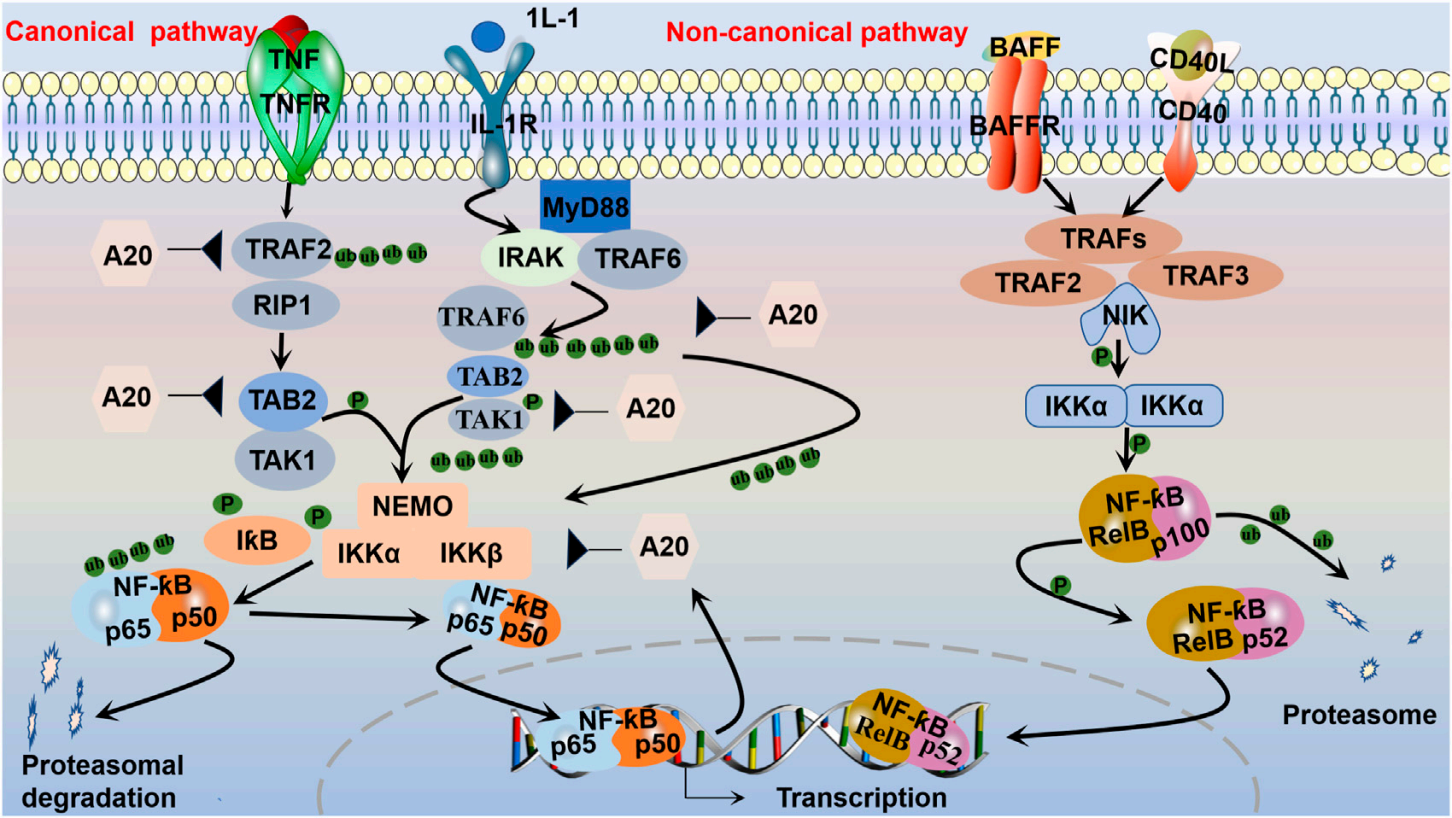
Canonical and non-canonical NF-ƙB pathways. The canonical pathway, which is triggered by TLRs and proinflammatory cytokines, such as TNF-α and IL-1, is activated by the kinase complex composed of IKKα, IKKβ, and regulatory NEMO, which are activated via TRAF complexes to ubiquitinate transforming growth factor beta-activated kinase (TAK1, TAB2). The activated IKKα-IKKβ-NEMO complex phosphorylates and leads to the ubiquitination of IƙB bound to NF-ƙB dimers (such as p50-p65). Then, the p50-p65 complex translocates to the nucleus to activate the transcription of target genes. After activation, the synthesized IƙB proteins and TRAF bind to and inhibit NF-ƙB activity and traffic it back to form a negative feedback loop. The non-canonical pathway is activated by receptors and their corresponding ligands, such as the ligand for CD40 (CD40L) and the ligand for BAFFR (BAFF). Inhibitory IKKα then phosphorylates p100, marking it for partial proteasomal degradation into p52. RelB-bound p52 is subsequently translocated to the nucleus to regulate gene transcription.

It is well established that NF-ƙB belongs to the Rel family of transcription factors, and there are five distinct members of the NF-ƙB family that share similar amino acid sequences: p50/p105 (NF-ƙB1), p52/p100 (NF-ƙB2), RelB, c-Rel, and p65 (RelA) (Xiao et al., 2001). NF-ƙB activation has been shown to play a role in promoting cell proliferation and regulating cell survival. It is generally observed that NF-ƙB possesses antiapoptotic properties. Notably, RelA-null mice exhibited significant TNF-mediated liver apoptosis. Both TNF and TNF-related apoptosis-induced ligand have been shown to activate concurrent death and NF-ƙB-dependent survival signals in renal cells. When NF-ƙB is inhibited, cell death is promoted. However, NF-ƙB/RelA activation is implicated in podocyte apoptosis in HIV-transgenic mice, which is mediated by NF-ƙB-dependent Fas and Fas ligand expression in nephrotoxin- and ischemia-induced tubular cell apoptosis (Sanz et al., 2010). The aggregation of activated NF-ƙB subunits into heterodimeric transcription factor complexes results in DNA-binding capacity and transactional activation potential. NF-ƙB, which is a heterodimer of p50 and p65, is a powerful activator of gene transcription (Song et al., 2019). NF-ƙB is an important signaling factor that regulates gene transcription and controls various processes, such as immunity, inflammation, cell growth, and apoptosis. The regulation of these genes is crucial for maintaining immune and inflammatory balance. Two different NF-ƙB pathways, the canonical and non-canonical NF-ƙB pathways, have different activation mechanisms (Lawrence, 2009) (Figure 2). Apparently, variations in NF-ƙB transcriptional activity are generated by multiple kinases phosphorylating the p50 subunit in response to various stimuli (Vermeulen et al., 2002). Activation of the canonical NF-ƙB pathway is mediated by the receptor, which does not require new protein synthesis and takes place within a few minutes, as opposed to activation of the non-canonical NF-ƙB pathway, which requires the synthesis of new proteins and is activated over a longer time period (Zarnegar et al., 2008). The release of various cellular stress factors, including cytokines such as TNF-α, IL-1, and growth factors, as well as neurotrophic factors or viral infections, promotes the cellular stress response by causing the transcription of certain genes that activate NF-ƙB (Singh and Singh, 2020). The inhibition of NF-ƙB entry into the nucleus or transcriptional alterations in the absence of stimulation by binding to inhibitor of kappa B (IƙB) maintains the inactive state of NF-ƙB dimers (Singh and Singh, 2020). The family of IƙB proteins consists of IƙBα, IƙBβ, and B-cell lymphoma-3; IƙBα can bind to heterodimeric (p50/Rel A) protein complexes (Yu et al., 2020). IƙBα can be divided into three structural domains: a 70-amino-acid N-terminal region, a 205-amino-acid internal region that is composed of ankyrin repeats, and a 42-amino-acid C-terminal region that contains a so-called PEST region. Mutation and protease sensitivity studies indicate that deletion of the N-terminal or C-terminal region does not inhibit the ability of IƙBα to interact with NF-ƙB. However, deletion of the C-terminus blocks the ability of IƙBα to inhibit the DNA binding of NF-ƙB. Mutations within the ankyrin repeat block interactions with NF-ƙB (Baldwin, 1996).

### 3.1 Canonical and non-canonical NF-ƙB signaling pathway

#### 3.1.1 Activation of the canonical NF-ƙB signaling pathway

Phosphorylation of the inhibitor of kappa B kinase (IKK) complex is the critical step in canonical NF-ƙB activation (Chen and Stark, 2019; Yu et al., 2020). Central to the activation of the canonical pathway is signal-induced IƙB phosphorylation by IKK. The IKK complex contains two kinase subunits, IKKα (IKK1) and IKKβ (IKK2), as well as the regulatory subunit IKKγ (NEMO) (Eluard et al., 2020). IKKβ modulates canonical pathway activation via phosphorylation of IƙB and requires the IKKγ subunit. By contrast, IKKα is essential for substitution pathway activation through the phosphorylation and processing of p100, which is isolated from both IKKβ and IKKγ (Sivandzade et al., 2019; Yu et al., 2020). IKKγ has no known intrinsic kinase activity but contains helix-loop-helix and leucine-zipper motifs that are known to be involved in protein-protein interactions. Without two IKKs or NEMO in MEFs, NF-ƙB activation is completely blocked after induction with various stimuli (Li and Verma, 2002). The IƙB protein keeps RelA and p50 away from the cytoplasm under stable conditions. By contrast, the inhibitor protein IƙB degrades IKK, allowing the transfer of heterodimers (e.g., p50/p65) from the cytosol to the nucleus, where they associate with DNA to enable the transcription of some genes encoding proinflammatory mediators (Song et al., 2019). This activation is rapid and transient, and the gene is simultaneously expressed with negative regulators such as IƙBα, p105, and A20, which are subsequently transported back to form a negative feedback loop (Blanchett et al., 2021). Deubiquitination is also essential for the inhibition of NF-ƙB. A20 inhibits NF-ƙB activity by deubiquitinating a few intermediary NF-ƙB signaling proteins, such as receptor-interacting protein 1 and tumor necrosis factor receptor associated factor. A20 is an important negative feedback regulator of NF-ƙB that is required for immune homeostasis. Early studies suggested that A20 may target the upstream receptor proximal molecule RIP1 in the TNF receptor and the ubiquitin-activating enzyme (E3) ligase TNF receptor-associated factor 6 in the IL-1 receptor signaling pathway to inhibit NF-ƙB activation (Pujari et al., 2013). During ubiquitination, the carboxy-terminal end of ubiquitin is covalently attached to a lysine in the target protein. Ubiquitination consists of a three-step enzymatic cascade initiated by the activation of ubiquitin by a ubiquitin-activating enzyme (E1) followed by the transfer of the activated ubiquitin to the active cysteine of a ubiquitin-conjugating enzyme (E2); ultimately, with the assistance of a ubiquitin-activating enzyme (E3) or ubiquitin ligase, the ubiquitin is transferred to a lysine on a substrate protein to form an isopeptide bond (Harhaj and Dixit, 2011). After the first ubiquitin molecule is added to a target protein, further ubiquitin molecules can attach to the first ubiquitin molecule, generating polyubiquitin chains (Napetschnig and Wu, 2013). The NF-ƙB pathway is negatively regulated by additional deubiquitinases in specific cells, including cylindromatosis, ovarian tumor domain deubiquitinase with linear linkage specificity (OTULIN), and cellular zinc finger anti-NF-ƙB (Cezanne) (Chen and Stark, 2019; Song et al., 2019).

#### 3.1.2 Activation of the non-canonical NF-ƙB signaling pathway

TNF cytokines and their corresponding TNF receptors activate atypical signaling pathways. Although alternative receptors are available to mediate the non-canonical NF-ƙB pathway, TNF receptors are commonly recognized. The TNF receptor family includes the lymphotoxin-β receptor, fibroblast growth factor-inducible factor 1, and B-cell activating factor receptor. NF-ƙB-inducible kinase (NIK) is activated by TNF or other relevant receptors, initiating the atypical pathway. In response to activation, the E3 ubiquitin ligase, which is a cellular inhibitor of apoptosis, degrades tumor necrosis factor receptor-associated factor 3 and contributes to NIK accumulation. When the accumulated levels are sufficient, NIK phosphorylates and activates IKKα in the IKK complex, and IKKα phosphorylates p100, a precursor subunit of NF-ƙB. Moreover, the SCF^bTrCP^ ubiquitin ligase complex phosphorylates p100, which is subsequently converted by the proteasome into p52, an NF-ƙB subunit with transcriptional functions. Genetic evidence has indicated that NIK is a core and specialized component of the non-canonical NF-ƙB pathway (Sun S. C., 2017).

### 3.2 Regulation of inflammation in renal fibrosis by the NF-ƙB signaling pathway

Inflammatory responses are characterized by the coordinated triggering of various pathways that regulate the expression of proinflammatory and anti-inflammatory mediators in resident tissue cells and blood-borne leukocytes (Lawrence, 2009). NF-ƙB has been proven to activate more than 500 genes that have been linked to inflammation-related responses (Gupta et al., 2010). NF-ƙB, which is a crucial regulator of inflammation and cell survival, is part of a classic pathway associated with the inflammatory response and a promising target for the diagnosis and treatment of kidney disease (Lawrence, 2009; Song et al., 2019). Inflammation is regulated at the molecular level by a variety of factors and molecules, including adhesion molecules such as intercellular adhesion molecules, chemokines such as monocyte chemoattractant protein 1 and interleukin-8, cytokines such as IL-6, TNF-α, and tumor necrosis factor-beta, proinflammatory enzymes such as cyclooxygenase-2 (COX-2) and 5-lipoxygenase, vascular endothelial growth factor, and the proinflammatory transcription factor NF-ƙB (Aggarwal, 2004). Among these mediators, NF-ƙB is a crucial regulator of inflammation (Ahn and Aggarwal, 2005). Peng et al. reported that autophagy-related protein 5 (ATG5) was essential for controlling inflammation during kidney fibrosis development. By limiting NF-ƙB signaling, ATG5 reduced the inflammatory response induced by wounded tubular epithelial cells in an autophagy-dependent manner (Peng et al., 2019). Garcinol suppressed NF-ƙB and proinflammatory cytokines such as TNF-α and IL-6, which preserved the nuclear expression of nuclear factor erythroid-derived 2-related factor 2 (Nrf2) and the levels of Nrf2-dependent antioxidants, including heme oxygenase-1 (HO-1), catalase, superoxide dismutase 1, and nicotinamide adenine dinucleotide phosphate quinone dehydrogenase 1 (NQO1), in tubulointerstitial fibrosis induced by non-diabetic unilateral ureteral obstruction (UUO). The administration of garcinol, a p300/CBP-associated factor (PCAF) inhibitor, effectively reversed the UUO-induced upregulation of total PCAF expression and histone 3 lysine 9 acetylation in the kidney. Additionally, it led to reductions in trichrome-positive staining areas, α-smooth muscle actin, and collagen. Furthermore, treatment with garcinol decreased the mRNA levels of TGF-β, matrix metalloproteinase-2, matrix metalloproteinase-9, and FN (Chung et al., 2019). These discoveries suggest that NF-ƙB is crucial for the progression of chronic inflammation in the kidney (Tak and Firestein, 2001).

## 4 The NF-ƙB signaling pathway in renal disease

**FIGURE 3:**
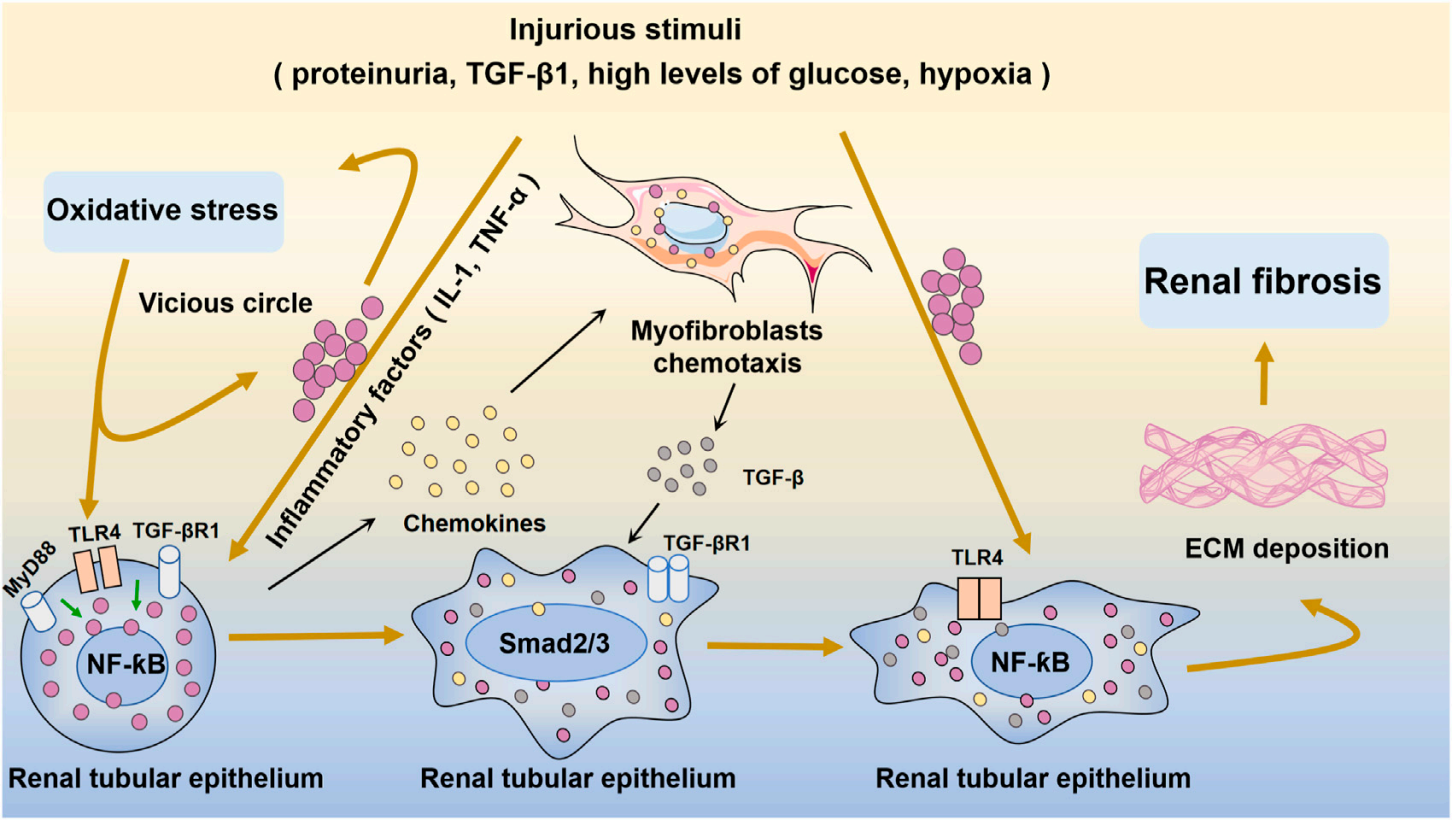
Oxidative stress and NF-ƙB-mediated inflammation in renal fibrosis and injury. Injurious stimuli trigger oxidative stress and inflammation and the secretion of inflammatory factors such as IL-1 and TNF-α. Inflammation activates TLR4 and stimulates quiescence in NF-ƙB in the renal tubular epithelium. Subsequently, excessive amounts of fibrogenesis-associated cytokines and growth factors, such as TGF-β, are produced. The chemokine gradient further drives cells to the site of injury. After injury, inflammatory cells infiltrate the renal tubular epithelium, and subsequent inflammatory cytokines activate myofibroblasts to proliferate and produce ECM. This eventually leads to renal fibrosis.

Aseptic inflammation, which occurs in the absence of infection or particular immunogens, plays an essential role in the inhibition of renal fibrosis (Lopez-De La Mora et al., 2015). The complex interplay between many intracellular signaling pathways influences the process of renal fibrosis. In the context of kidney damage, NF-ƙB is a significant transcriptional driver of inflammation and fibrosis (Reid and Scholey, 2021) (Figure 3). UUO is a classic model of renal fibrosis. Activation of the NF-ƙB signaling pathway has been demonstrated in the obstructed kidneys of UUO mice (Wang et al., 2020). Inflammation, including macrophage infiltration and the generation of proinflammatory cytokines, is the primary etiology of renal interstitial fibrosis following ureteral obstruction (Chevalier et al., 2009). Ongoing inflammation can lead to gradual kidney damage and eventually permanent renal parenchymal damage (Xianyuan et al., 2019).

### 4.1 The NF-ƙB signaling pathway in AKI

AKI is a condition with a wide range of pathologies (Siew and Davenport, 2015; Ma et al., 2023). AKI is characterized by a rapid decrease in kidney function and a high level of morbidity and mortality. Sepsis, ischemia-reperfusion injury (IRI), and nephrotoxicity (radiographic agents and non-steroidal anti-inflammatory drugs) are the main factors involved in the development of AKI (Amrouche et al., 2017; Wang J. et al., 2022). Increasing evidence suggests that AKI-associated renal inflammation can be sustained even following the remission of acute injury and the recovery of renal function, resulting in the slow progression of renal fibrosis (Andrade-Oliveira et al., 2019). AKI etiologies are categorized as prerenal, intrinsic renal, or postrenal. Type 1 cardiorenal syndrome and hepatorenal syndrome decrease the effective circulating volume and increase central venous pressure, further leading to renal dysfunction and the development of prerenal AKI. If early intervention is not timely, renal parenchymal ischemia worsens, and renal tubular cell damage in prerenal AKI can further develop into AKI (Turgut et al., 2023). By contrast, AKI is an ischemic injury to the kidney, and the etiology of AKI can be divided into glomerular, tubulointerstitial, and vascular diseases. Among these conditions, acute tubular necrosis (ATN) is the major known cause of intrarenal AKI, which can include renal ischemia and nephrotoxicity and can cause harm to tubular endothelial and epithelial cells, leading to the development of ATN. Ischemic ATN is caused by persistent hypotension, hypovolemia, and low renal irrigation and is most common in patients with severe hypotension. Postrenal AKI is mainly caused by obstruction; 5%–10% of AKI cases are caused by urinary tract obstruction and it is more common in elderly individuals, and the most common types of obstruction are prostate cancer, prostate hypertrophy, cervical cancer, and retroperitoneal disease (Mercado et al., 2019).

AKI causes inflammation that exacerbates kidney damage, and controlling inflammation has been shown to mitigate further deterioration of kidney injury and promote slow recovery (Bonventre and Zuk, 2004). The systematic regulation of the NF-ƙB pathway, an important component of AKI pathogenesis, can influence the severity of AKI (Song et al., 2019). A previous study showed that calcium dobesilate mitigated renal impairment and inflammation in sepsis-associated AKI by downregulating the NF-ƙB signaling pathway in lipopolysaccharide (LPS)-induced mice (Xie et al., 2022). Additional studies have suggested that inhibiting miR-494 by blocking the NF-ƙB pathway, which reduces apoptosis in renal tubular epithelial cells, could attenuate the inflammatory response and oxidative stress in the kidney tissues of rats with LPS-induced AKI (Lu et al., 2022). Fisetin, a naturally occurring flavonoid, reduces inflammation in the kidney and apoptosis in mice with LPS-induced septic AKI by inhibiting the Src-mediated NF-ƙB p65 signaling pathway (Ren et al., 2020). Additionally, NF-ƙB can induce COX-2 expression, which is pivotal in the development of AKI (Ranganathan et al., 2013). Thalidomide attenuates glycerol-induced AKI in rats by inhibiting NF-ƙB and COX-2 (Amirshahrokhi, 2021). In addition, IRI is a common factor in the AKI-to-CKD transition and renal fibrosis. Postischemic fibrosis involves the AKI-to-CKD transition and is subsequently influenced by oxidative stress and inflammation.

### 4.2 The NF-ƙB signaling pathway in CKD

CKD is a progressive disease that affects between 8% and 16% of the global population and is a major cause of death (Chen T. K. et al., 2019; Rashid et al., 2022). A glomerular filtration rate less than 60 mL/min/1.73 m^2^, an albuminuria level of at least 30 mg/24 h, or symptoms of kidney disease (such as hematuria or structural abnormalities such as polycystic or dysplastic kidneys) that persist for more than 3 months are considered indicators of kidney damage (Kalantar-Zadeh et al., 2021; Zhao, 2022). Multiple factors are involved in the inflammatory state of CKD, including oxidative stress, increased production of proinflammatory cytokines, dysbiosis of the gut flora, altered adipose tissue metabolism, acidosis, and chronic and recurrent infections (Rapa et al., 2019; Yang and Wu, 2021; Zhao, 2022; Liu et al., 2023). Oxidative stress and inflammation play significant roles in the pathophysiology of CKD. Oxidative stress and inflammation strongly interact to create a vicious cycle, and oxidative stress triggers inflammation through a number of processes, including the transcriptional activation of NF-ƙB, leading to immune cell activation and recruitment. Oxidative stress mediates inflammation through the generation of proinflammatory oxidized lipids, advanced oxidation protein products, and AGEs (Duni et al., 2019). Conversely, inflammation induces oxidative stress through the production of ROS and nitrogen species through the activation of leukocytes and resident cells. These incidents enhance renal injury by causing renal necrosis and fibrosis (Chen et al., 2017). Moreover, various studies have substantiated the anti-inflammatory effects of Nrf2 by inhibiting the expression of inflammatory genes, including those responsible for the TNF-α-induced production of monocyte chemoattractant protein-1 (MCP-1) and vascular cell adhesion molecule-1. Dysfunctional Nrf2 activation, which is observed in CKD, renders the kidneys susceptible to the impacts of oxidative stress. Furthermore, this dysfunctional activation exacerbates intrarenal inflammation by mediating the accumulation of hydroperoxides and lipoperoxides, which are potent activators of NF-ƙB (Duni et al., 2019). Globally, the most commonly reported causes of CKD are diabetes and/or hypertension, but other conditions, such as cardiovascular disease and kidney transplantation, can lead to CKD development (Luyckx et al., 2020). Next, we summarize the effect of the NF-ƙB signaling pathway on renal fibrosis in CKD (immunoglobulin A nephropathy, membranous nephropathy, diabetic nephropathy, hypertensive nephropathy, and kidney transplantation).

#### 4.2.1 Immunoglobulin A nephropathy (IgAN)

Glomerular diseases present as a spectrum of clinical syndromes and include various glomerular cell types, such as mesangial cells (MCs), endothelial cells, podocytes, parietal epithelial cells, and infiltrating inflammatory cells (Scindia et al., 2010). Glomerular disease is characterized by inflammation, and activation of the NF-ƙB and TGF-β signaling pathways is important for disease progression. Immunoglobulin aggregates activate MCs by signaling through surface Fc receptors. Mesangial alterations observed after glomerular injury involve the production of chemoattractants for inflammatory cells, the proliferation of MCs, the loss of mesangial matrix (mesangiolysis), and the excessive production of ECM, which leads to mesangial expansion (Scindia et al., 2010). Notably, drugs that target the NF-ƙB pathway in animal models have renoprotective effects, indicating that these agents may be viable treatments for glomerulopathy. IgAN is the most prevalent primary glomerulonephritis in the world (Möller-Hackbarth et al., 2021). Currently, IgAN is considered an immune-mediated disease characterized by immunoglobulin A deposition in the mesangial area (Coppo and Amore, 2004). The deposition of IgA triggers MC proliferation and monocyte/macrophage infiltration, resulting in the activation of the complement system. Subsequently, activated MCs release ECM proteins, growth factors, and proinflammatory cytokines (Apeland et al., 2020). IgAN is identified in approximately 50% of kidney biopsies in Asia and up to 45% of biopsies in China (Zhang J. et al., 2020). In particular, 30%–40% of patients with IgAN progress to ESRD within 20 years (Bai et al., 2019). A study showed the levels of 37 different inflammation-related factors in IgAN patients with stage 1–4 CKD. The IgAN group had elevated levels of nine inflammation-related factors according to univariate analysis. However, multivariate analysis revealed five important factors that were characteristic of patients with IgAN: osteopontin, a proinflammatory mediator, proliferation-inducing ligand, matrix metalloproteinase-3, TNF receptor-1, and TNF-like weak inducer of apoptosis, which are members of the TNF receptor-2 non-canonical NF-ƙB pathway. This pathway may be activated in a slow and persistent manner, and dysregulated pathway activation contributes to the pathogenesis of various inflammatory diseases (Sun S.-C., 2017). Apolipoprotein C1 is thought to be a central secretory gene associated with IgAN, and mechanistic studies have indicated that knockdown of apolipoprotein C1 can ameliorate IgAN and renal fibrosis by inhibiting the NF-ƙB pathway (Yu et al., 2023). In addition, a study revealed the increased expression of toll-like receptor 4 (TLR4) in mesangial cells, which subsequently activated the MyD88-NF-ƙB signaling pathway. This signaling cascade ultimately results in the production of proinflammatory cytokines such as TNF-α, IL-6, and MCP-1, contributing to kidney damage in secretory IgA-mediated human renal mesangial cells (Zhang J. et al., 2020).

#### 4.2.2 Membranous nephropathy

The most prevalent form of adult nephrotic syndrome, which is known as membranous nephropathy (MN), is a primary glomerular disease that develops following kidney transplantation (Hua et al., 2023; Li et al., 2023). In approximately 80% of patients, MN has no root cause (primary MN), and 20% of cases are related to drugs or other diseases, such as malignancy, hepatitis virus infection, or systemic lupus erythematosus (Ronco et al., 2021; Zhao et al., 2023). MN patients are diagnosed by the presence of the phospholipase A_2_ receptor before they progress to renal failure (Wang et al., 2022b; Salvadori and Tsalouchos, 2022). However, the subepithelium-like immunocomplex deposit-mediated downstream molecular pathways are poorly understood (Miao et al., 2023a; Miao et al., 2023b). A recent study suggested that patients with primary MN exhibited significant upregulation of CD3, NF-ƙB p65, and COX-2 protein expression and significant downregulation of Nrf2 and HO-1 protein expression in kidney tissues (Wang et al., 2023b), indicating activation of the NF-ƙB signaling pathway and impairment of the Nrf2 signaling pathway. This was further demonstrated by the significant upregulation of the protein expression of the phosphorylated inhibitors kappa B alpha, NF-ƙB p65, and downstream gene products, including COX-2, MCP-1, inducible nitric oxide synthases, 12-lipoxygenase, p47^phox^, and p67^phox^; additionally, the protein expression of Nrf2 and its downstream gene products, including HO-1, catalase, the glutamate-cysteine ligase catalytic subunit, glutamate-cysteine ligase modifier subunit, manganese superoxide dismutase, and NQO1, was significantly downregulated in the kidney tissues of CBSA-induced rats (Wang et al., 2023b). These results were further verified in zymosan activation serum (ZAS)-stimulated podocytes (Wang et al., 2023b). By contrast, treatment with the NF-ƙB inhibitor BAY 11-7082 and NF-ƙB p65 siRNA inhibited the protein expression of NF-ƙB p65 and COX-2 and preserved the protein expression of Nrf2 and HO-1 in ZAS-induced podocytes (Wang et al., 2023b). Tian et al. reported that CBSA injection led to the deposition of C3 and immunoglobulin G and reduced the protein expression of podocin and synaptopodin, which are associated with the NF-ƙB signaling pathway (Miao et al., 2022b). The NF-ƙB signaling pathway plays an important role in immune modulation. Previous studies revealed that the NF-ƙB pathway is involved in the pathogenesis of MN (Wang et al., 2022b). These findings suggest the activation of oxidative stress and inflammation in MN.

#### 4.2.3 Diabetic nephropathy (DN)

Diabetes mellitus occurs regardless of age, sex, ethnicity, education level, or economic status. Up to 20% of diabetic patients develop diabetic nephropathy (DN) (Hung et al., 2021; Rangel et al., 2021). DN is characterized by a thickened basal lamina, renal fibrosis, proteinuria, and the accumulation of mesangial cells (Wang H. et al., 2021). DN is related to hyperglycemia-induced metabolic changes leading to glomerular hypertrophy, glomerulosclerosis, tubulointerstitial inflammation and fibrosis, and renal remodeling, which includes glomerular and tubular hypertrophy, inflammation, and extracellular matrix accumulation (Sun et al., 2020; Wang and Chen, 2022). One of the main pathological factors in renal fibrosis linked to DN is thought to be inflammation. Infiltrating cells are also a source of cytokines and other mediators that promote the onset and progression of renal injury, as well as amplify and perpetuate a preexisting inflammatory response (Samsu, 2021). Under diabetic pathological conditions, high levels of glucose induce cells to generate massive amounts of ROS, thereby activating multiple downstream inflammatory signaling pathways and leading to the induction and accelerated development of inflammatory fibrosis (Zhuang et al., 2020). After hyperglycemic therapy, TLR4 activates high mobility group box 1, which in turn mediates the development of tubulointerstitial inflammation in DN patients. Additionally, TLR4 participates in the production of TRAF and IL-1 receptor-related kinase via myeloid differentiation factor 88, which eventually results in the production of NF-ƙB and mediates the development of DN (Zhou et al., 2021). Notably, NF-ƙB is involved in DN by regulating inflammatory factors, chemokines, and cell adhesion proteins (Navarro-González et al., 2011). By preventing the activation of the NF-ƙB pathway in a rat model of DN, silencing paternally expressed gene 3 could minimize renal fibrosis in DN (Guan et al., 2020). The NF-ƙB signaling pathway is involved in DN.

#### 4.2.4 Hypertensive nephropathy

Hypertension and CKD are strongly related to pathophysiological states, and sustained hypertension leads to the deterioration in renal function and an ongoing decrease in renal function, which leads to the deterioration in blood pressure control (Abraham et al., 2022). Chronic arterial hypertension can cause the development of renal-angiosclerosis, which is a critical cause of ESRD, and CKD might be aggravated by arterial hypertension due to different pathogenetic mechanisms, such as sympathetic hyperactivity, endocannabinoid dysfunction, and volume overload (Cice et al., 2020). According to previously published studies, the levels of C-reactive protein, TNF-α, IL-6, MCP-1, plasminogen activator inhibitor-1, and adhesion molecules are elevated in individuals with hypertension (Montecucco et al., 2011). It has been reported that H_2_S administration alleviates IRI by decreasing local and systemic intercellular adhesion molecule expression and NF-ƙB levels in the kidneys of normotensive and L-nitro-arginine-methyl-ester (L-NAME)-induced hypertensive rats (Hashmi et al., 2021). These findings indicate the involvement of inflammation in hypertension.

#### 4.2.5 Kidney transplantation

A consistent decrease in the glomerular filtration rate is linked to the progression of CKD to ESRD (Wishahi and Kamal, 2022). Stage 5 CKD and ESRD patients require peritoneal or programmed hemodialysis or renal transplantation. Renal transplantation results in better survival for ESRD patients than dialysis treatment (Gadelkareem et al., 2023). Renal transplantation provides a better quality of life and better affordability (Cabezas et al., 2022). However, the majority of transplants experience chronic renal allograft dysfunction (CAD), which negatively impacts long-term graft survival (Zhou et al., 2022). CAD, which was previously mentioned as chronic allograft nephropathy, is a polyfactorial disease related to the progression of renal interstitial fibrosis (Gui et al., 2021). Under certain circumstances, grafts may exhibit dysfunction regardless of the immunosuppressive regimen used. The local and systemic proinflammatory state in patients worsens with the development of renal graft failure. According to the Banff classification, significant pathomorphological features of most variants of this dysfunction can reflect the molecular mechanisms involved in tissue stress that leads to fibrin formation and inflammation (Gusev et al., 2021). Kidney transplants unavoidably experience ischemia once they have been removed from the donor. Ischemia is regarded as an unavoidable event after kidney transplantation. IRI reduces the prolongation of graft survival, which has been recognized as an unavoidable event after renal transplantation. Early after surgery, IRI can lead to end-stage graft loss through a reduction in renal function clumps, which can cause graft vascular injury, chronic hypoxia, and subsequent fibrosis (Zhao et al., 2018). Consequently, an effective strategy to alleviate the IRI-induced inflammatory response and oxidative stress injury during postrenal transplantation is needed.

## 5 Therapeutic strategies targeting the NF-ƙB signaling pathway in renal fibrosis

In the clinical setting, treatments for renal fibrosis are ineffective or unsafe (Breyer and Susztak, 2016). Although numerous therapeutic strategies to prevent and treat renal fibrosis have been explored, the prognosis of patients is still poor (Salvadori and Tsalouchos, 2022). Thus, there is an urgent need for the development of specific and efficacious antifibrotic drugs to improve AKI and CKD (Tanemoto and Mimura, 2022; Allinson et al., 2023; Habshi et al., 2023; Song and Gong, 2023). Accordingly, renal fibrosis can be efficiently treated by inhibiting the NF-ƙB signaling pathway. Regardless of the underlying etiology, various strategies have been used to protect against renal fibrosis by targeting the NF-ƙB signaling pathway in this context (Table 1).

**TABLE 1:** Summary of small molecular inhibitors of NF-ƙB signaling in renal fibrosis.

Agents	Targets	Outcomes	References
Metformin	NF-ƙB pathways	Reliving the processes of inflammation and fibrosis in individuals with DN	Zhou et al. (2021)
Telbivudine	NF-ƙB pathways	Reducing renal fibrosis and inflammation	Chen and Li (2018)
Tacrolimus	NF-ƙB pathways	Suppressing intrarenal inflammation	Kim et al. (2021)
Bortezomib	NF-ƙB-TNF-α-Akt-mTOR-P70S6K-Smurf2 pathway	Improving renal allograft tubulointerstitial fibrosis	Suo et al. (2021)
Pioglitazone	NF-ƙB pathways	Suppressing renal IRI-induced inflammation	Zou et al. (2021)
5-MTP	NF-ƙB and Nrf2 pathways	Attenuating tubulointerstitial fibrosis	Chen et al. (2019a)
TPH-1	NF-ƙB and Nrf2 pathways	Exacerbating renal injury and fibrosis	Chen et al. (2019a)
Shenkang injection	NF-ƙB and Nrf2 pathways	Ameliorating renal fibrosis by inhibiting oxidative stress and inflammation	Xu et al. (2016)
Chrysophanol	NF-ƙB pathways	Improving renal fibrosis	Gu et al. (2022)
*Poria cocos*	NF-ƙB pathway, COX-2, MCP-1, HO-1, catalase and NQO1	Ameliorating renal fibrosis	Feng et al. (2019a)
Poricoic acid A	Gas6-Axl-NF-ƙB-Nrf2 pathway	Inhibiting AKI-to-CKD transition	Chen et al. (2019b)
Ergone *Polyporusumbellatus*	NF-ƙB and Nrf2pathways	Ameliorating tubulointerstitial fibrosis	Chen et al. (2019c)
	NF-ƙB and Nrf2 pathways	Ameliorating tubulointerstitial fibrosis	Chen et al. (2019c)
Isoliquiritigenin	NF-ƙB pathway	Lowering kidney inflammation and fibrosis	Liao et al. (2020)
Artemisinin	NF-ƙB pathway	Attenuating tubulointerstitial inflammation and fibrosis	Wen et al. (2019)
Ferulic acid	NF-ƙB pathway	Reduced podocyte damage	Qi et al. (2020)
Quercetin	Mincle-Syk-NF-ƙB pathway	Lessening AKI-induced kidney inflammation and damage	Tan et al. (2020)
Red ginseng	NF-ƙB and PI3K-Akt pathways	Improving inflammation and the oxidative stress response	Li et al. (2019)
Zhen-wu-tang	NF-ƙB pathway and NLRP3 inflammasome	Inhibiting kidney inflammation and improving podocyte injury and structure in MN rats	Liu et al. (2019)
Baicalin	TLR4-NF-ƙB pathway	Retarding renal fibrosis	Zhang et al. (2020b)
MiR-21-5p	NF-ƙB pathway	Progressing renal fibrosis and enhancing renal inflammation	Liu et al. (2021)
MiR-103a-3p	NF-ƙB p65 pathway	Leading to renal inflammation and fibrosis	Lu et al. (2019)
LncRNAKCNQ1OT1	NF-ƙB pathway	Modulating DN cell proliferation, apoptosis, and fibrosis	Jie et al. (2020)

### 5.1 Chemical agents for treating renal fibrosis

For more than 50 years, metformin has been used as an antihyperglycemic medication with few negative side effects. More recently, advances have shown its antifibrotic effects on several organs, such as the kidney, liver, and other tissues (Sun et al., 2022). Glomerular tenascin-C (TNC) levels in DN rats were measured, and the effect of TNC expression on inflammatory and fibrogenic factors was examined in high glucose-cultured rat mesangial cells. After administering an miR-155-5p inhibitor to selectively suppress the expression of miR-155-5p for 6 h, the culture medium was replaced, and the cells were incubated with normal or high glucose for 24 h. This intervention reduced the levels of the fibrogenic factors FN and CTGF. Following the targeted silencing of TNC expression, there was a decrease in TLR4 expression and the phosphorylation of the inflammatory factor NF-ƙB p65. Additionally, miR-155-5p expression was downregulated, and the expression of the fibrotic factors CTGF and FN was decreased in high-glucose-treated rat mesangial cells (Zhou et al., 2021). Compared with normal rats, diabetic rats had significantly higher serum TNC levels. The levels of phosphorylated NF-ƙB p65 and miR-155-5p were significantly decreased when TNC was downregulated, indicating that TNC controlled miR-155-5p expression via the NF-ƙB signaling pathway, which controls inflammation and fibrosis in DN rats. Metformin treatment may relieve inflammation and fibrosis in individuals with DN by reducing the protein levels of TNC, p-NF-ƙB p65, CTGF, and FN (Zhou et al., 2021). Additionally, telbivudine is an orally bioavailable L-nucleoside with potent and specific antiviral activity against the hepatitis B virus, and it has been used widely to treat chronic HBV infection. Clinical findings indicate that telbivudine therapy improves renal function. A study showed that the UUO group had decreased levels of the inhibitor IƙBα and increased levels of p-IƙBα compared with those in the control group. In the telbivudine-treated group, TLR4 activation was inhibited; IKKα, p-IKKα, and p-IƙBα expression was significantly attenuated; and the decrease in IƙBα was reversed to a level that was significantly higher than that in UUO rats. These findings indicated that the NF-ƙB signaling pathway allows telbivudine to reduce renal fibrosis and inflammation in UUO patients (Chen and Li, 2018). Nifuroxazide is a safe nitrofuran antibacterial drug that is used clinically to treat acute diarrhea and infectious traveler’s diarrhea or colitis. Recent studies revealed that nifuroxazide has multiple pharmacological effects, including anticancer, antioxidant, and anti-inflammatory effects (Althagafy et al., 2023). Nifuroxazide decreased the levels of proinflammatory cytokines, including TGF-β1, TNF-α, IL-1β, and MCP-1, and macrophage infiltration in a UUO rat model. Additionally, it improved renal function, decreased tissue injury and fibrosis, and reduced renal oxidative damage and inflammation, which were associated with inhibiting the NF-ƙB signaling pathway (Hassan et al., 2021). In a classical model of lupus nephritis, the administration of MRL/lpr, which are hydrophobically modified glycol chitosan nanomicelles loaded with tacrolimus, decreased glomerulosclerosis and suppressed intrarenal inflammation via the NF-ƙB signaling pathway (Kim et al., 2021). Suo et al. (2021) reported that bortezomib improved renal allograft tubulointerstitial fibrosis by inhibiting the NF-ƙB-TNF-α-Akt-mTOR-P70S6K-Smurf2 pathway via IƙBα protein stabilization. Suo et al. reported that TNF-α and MCP-1 expression was reduced in the renal tissue of rats with renal IRI, indicating that pioglitazone suppressed the renal IRI-induced inflammatory response by inhibiting the NF-ƙB signaling pathway (Zou et al., 2021).

Our previous studies revealed that 5-methoxytryptophan (5-MTP) levels strongly correlated with clinical marker levels in patients with progressive CKD (Chen et al., 2019a). The level of 5-MTP decreased with the progression of CKD and in the obstructed kidneys of UUO mice. Treatment with 5-MTP slowed tubulointerstitial fibrosis, inhibited the NF-ƙB signaling pathway, and enhanced the Nrf2 signaling pathway in mice with UUO or IRI, as well as in HK-2 cells (Chen et al., 2019a). Our examination of the biological effects of 5-MTP on UUO mice, as well as HK-2 cells and human mesangial cells, revealed that 5-MTP mitigated the proinflammatory factor NF-ƙB p65. Additionally, it decreased the expression of its target gene products, MCP-1 and COX-2, while enhancing the expression of the anti-inflammatory and antioxidant transcription factor Nrf2. Furthermore, there was an increase in the expression of its target gene products, HO-1 and NQO-1 (Chen et al., 2019a). Tryptophan hydroxylase-1 (TPH-1) is a key enzyme involved in 5-MTP synthesis. TPH-1 overexpression ameliorated renal damage by suppressing renal inflammation and fibrosis, whereas TPH-1 deficiency exacerbated renal injury and fibrosis by activating NF-ƙB and inhibiting the Nrf2 signaling pathway (Chen et al., 2019a). Our results indicated that TPH-1 could be a target in the treatment of CKD. Collectively, these findings suggest that the NF-ƙB signaling pathway is a critical therapeutic target for the treatment of renal fibrosis.

### 5.2 Natural products for treating renal fibrosis

Many publications have demonstrated that natural products have beneficial effects on renal fibrosis and that natural products are a promising source of new medicines (Newman and Cragg, 2020; Wang et al., 2023a). A growing body of evidence has revealed the molecular mechanisms of natural products, and numerous natural products suppress renal fibrosis though the NF-ƙB pathway (Chen et al., 2018). With the advantages of multiple pathways, multiple targets, and few side effects, Chinese medicine compounds have the potential to be used in therapeutic and adjunctive therapy for nephrogenic fibrosis. According to Chinese medicine, kidney deficiency and blood stasis are not isolated but are interrelated and coexist; kidney deficiency must be accompanied by blood stasis, and blood stasis exacerbates kidney deficiency. As a result, kidney deficiency and blood stasis coexist throughout the course of the disease. The main mechanism of CKD and the root cause of renal fibrosis is the “interior retention of damp heat and toxin stasis.” Shenkang injection is widely used to treat patients with CKD (Wang et al., 2022c). Our study demonstrated that Shenkang injection and its main components, chrysophanol, emodin, and rhein, ameliorated renal fibrosis by inhibiting oxidative stress and inflammation by improving the NF-ƙB and Nrf2 signaling pathways in adenine-induced CKD rats (Xu et al., 2016). In addition, Gu et al. showed that chrysophanol improved renal fibrosis by regulating the NF-ƙB pathway (Gu et al., 2022). *Poria cocos* is a well-known medicinal mushroom that is widely used in Asia and North America. *Poria cocos* has diverse biological activities, such as anti-inflammatory, antioxidant, antitumor, and lipid-lowering effects. Our previous study demonstrated that *Poria cocos* extracts ameliorated renal fibrosis by regulating redox signaling, as indicated by the downregulated protein expression of NF-ƙB and its downstream target gene products (*COX-2* and *MCP-1*) and the upregulated protein expression of Nrf2 and its downstream target gene products (*HO-1*, catalase, and *NQO1*) in 5/6 nephrectomized rats (Feng et al., 2019a). Similarly, our study showed that poricoic acid ZM and poricoic acid ZP isolated from the surface layer of *Poria cocos* ameliorated renal fibrosis by regulating the NF-ƙB and Nrf2 signaling pathways in TGF-β1-induced HK-2 cells and UUO mice (Wang et al., 2020). Poricoic acid A is a major component of the surface layer of *Poria cocos*. Our previous study demonstrated that poricoic acid A inhibited the AKI-to-CKD transition by regulating the Gas6-Axl-NF-ƙB-Nrf2 signaling cascade in IRI rats (Chen et al., 2019b). *Polyporus umbellatus* is commonly used for its diuretic activity and to treat renal disease (Zhao Y. Y. et al., 2009; Zhao, 2013). Ergone, one of the main components of *Polyporus umbellatus* (Zhao Y. et al., 2009; Zhao et al., 2010a; Zhao et al., 2010b), has been shown to slow renal fibrosis (Zhao et al., 2011; Chen et al., 2016). Our previous study demonstrated that the extracts of *Polyporus umbellatus* and ergone ameliorated tubulointerstitial fibrosis by activating IƙBα/NF-ƙB, which was accompanied by the significant upregulation of inflammatory genes, including *MCP-1* and *COX-2*, and the downregulation of genes in the antioxidant system, including Nrf2 and its downstream gene products (including *HO-1*, catalase, and *NQO1*) (Chen L. et al., 2019).

Isoliquiritigenin is a chalcone flavonoid found in licorice and shallots. Isoliquiritigenin has anti-inflammatory, antifibrotic, and antitumor properties. Treatment with isoliquiritigenin improved UUO-induced renal dysfunction by significantly downregulating the mRNA expression and secretion of IL-1β, IL-6, TNF-α, and MCP-1, inhibiting the phosphorylation of Syk and NF-ƙB and reducing the expression of α-SMA and Col III *in vitro and in vivo*. Isoliquiritigenin decreased kidney inflammation and fibrosis by suppressing the NF-ƙB signaling pathway in UUO mice (Liao et al., 2020). Artemisinin is known as the most powerful drug for treating malaria. Clinical research has suggested that artemisinin possesses anti-inflammatory and immunomodulatory characteristics in addition to its antimalarial effect. Artemisinin plays a protective role in attenuating renal tubulointerstitial inflammation and fibrosis by reducing the protein levels of fibrosis markers, such as TGF-β1 and CTGF, by inhibiting the NF-ƙB pathway in 5/6 nephrectomized rats (Wen et al., 2019). Ferulic acid is a phenolic compound that exists in both fruits and plants and has various pharmacological activities, such as regulating blood glucose and blood lipids and antioxidant, anti-inflammatory, and antifibrotic activities. Furthermore, prolonged administration of ferulic acid significantly downregulated the protein expression of p-NF-ƙB p65, TNF-α, TGF-β1, and collagen IV. Moreover, it upregulated the expression of the nephrin and podocin proteins in renal tissues by inhibiting the NF-ƙB signaling pathway in DN rats (Qi et al., 2020). Quercetin is a common flavonoid that is abundant in the leaves, stems, and fruits of several plant species. It has a variety of biological activities, such as antioxidant, anticancer, and anti-inflammatory effects. Tan et al. reported that quercetin significantly ameliorated the serum levels of creatinine, IL-1, IL-6, and TNF-α and reduced inflammatory factor production by inhibiting Mincle-Syk-NF-ƙB signaling axis-mediated macrophage inflammation, consequently decreasing AKI-induced kidney inflammation and damage (Tan et al., 2020). Red ginseng possesses a variety of biological benefits, including the ability to prevent tumor formation, lower blood sugar levels, and increase antioxidant activity. 1-Aminyl-fructosyl-glucose is a significant and typical non-saponin component of red ginseng. Tan et al. demonstrated that treatment with 1-arginine-fructosyl-glucose improved cisplatin-induced acute kidney injury by inhibiting oxidative stress, NF-ƙB-mediated inflammation, and PI3K-Akt-induced apoptotic signaling pathways (Li et al., 2019). Tan et al. showed that Zhen-wu-tang, a Chinese compound formula, has good therapeutic effects on MN by enhancing kidney function in an MN rat model triggered by cationic bovine serum albumin, inhibiting kidney inflammation and improving podocyte injury and structure, which was associated with inhibiting the NF-ƙB pathway and NLRP3 inflammasome (Liu et al., 2019). In addition, baicalin protected against renal fibrosis by augmenting miR-124 and silencing the downstream TLR4-NF-ƙB pathway in streptozotocin-induced DN mice (Zhang S. et al., 2020). Therefore, baicalin may serve as a renoprotective agent. There are a variety of natural products. Further in-depth studies of the underlying mechanism by which natural products affect the NF-ƙB signaling pathway would be beneficial for the popularization and application of natural products.

### 5.3 Targeting non-coding RNAs to treat renal fibrosis

MicroRNAs (miRNAs), which are a large family of small and highly conserved non-coding RNAs, regulate gene expression through translational repression or mRNA degradation (Zou et al., 2017). MiRNAs are RNAs of approximately 22 nucleotides in length that drive the post-transcriptional repression of target mRNA in a variety of eukaryotic lineages (Bartel, 2018). The dysregulation of miRNAs is associated with many disorders, including renal fibrosis (Chung and Lan, 2015). MiR-21 is a frequently referenced miRNA in the renal fibrosis field. It was found to be dysregulated in several renal fibrosis models and human specimens. A study revealed that an increase in miR-21-5p expression levels in ureteral obstruction led to ECM deposition and progressive renal fibrosis by targeting sprouty receptor tyrosine kinase signaling antagonist 1, which activated the NF-ƙB signaling pathway and enhanced renal inflammation (Liu et al., 2021). Lu et al. reported that angiotensin II increased circulating miR-103a-3p levels, which reduced serine/threonine-protein kinase levels in glomerular endothelial cells, resulting in the overactivation of NF-ƙB p65 and leading to renal inflammation and fibrosis (Lu et al., 2019). Therefore, these findings suggest that microRNAs are associated with inflammation in renal injury.

Research has shown that long non-coding RNAs (lncRNAs) are closely related to the development and prognosis of renal fibrosis (Jia et al., 2020). LncRNAs are more than 200 nucleotides in length and do not encode proteins in most instances (Wang Y. N. et al., 2021). LncRNAKCNQ1OT1 is an lncRNA, and studies have shown that KCNQ1OT1 is upregulated in DN patients, human glomerular mesangial cells (HGMCs), and human renal glomerular endothelial cells (HRGECs) in response to high glycemic induction. Treatment with 30 mM glucose to simulate DN conditions resulted in an increase in KCNQ1OT1 in both HGMCs and HRGECs compared with that in the normal group. Moreover, in the high glucose group, there was a significant increase in cell viability and a notable decrease in the rate of apoptosis compared with those in the normal group. These findings indicate that KCNQ1OT1 promotes DN progression. MTT assays also showed that KCNQ1OT1 knockdown suppressed proliferation in both HGMCs and HRGECs. Apoptosis was promoted in HGMCs and HRGECs transfected with siKCNQ1OT1, and this effect was reversed by co-transfection with pcDNA-SORBS2. In addition, the expression levels of the fibrosis-related proteins FN, Col-4, and TGF-β1 were markedly downregulated by transfection with siKCNQ1OT1, and overexpression of SORBS2 reversed the changes in fibrosis-related proteins caused by KCNQ1OT1 knockdown. Moreover, the mRNA and protein levels of NF-ƙB were decreased in HGMCs. In brief, KCNQ1OT1 knockdown inhibited cell proliferation and fibrosis and induced apoptosis, suggesting that KCNQ1OT1 modulated DN cell proliferation, apoptosis, and fibrosis through the NF-ƙB signaling pathway (Jie et al., 2020).

In a clinical setting, CKD patients receiving hemodialysis were treated with curcumin for 3 months, and inflammatory markers, NF-ƙB mRNA expression, and high-sensitivity C-reactive protein levels decreased. This finding suggested that oral curcumin may have anti-inflammatory effects on these patients (Alvarenga et al., 2020). A study enrolled patients with stage 3 to 5 CKD into α-ketoacid tablet and non-α-ketoacid tablet groups according to their medications. After adjusting for basic demographic factors, the rate of the decrease in the eGFR in stage 4 and stage 5 patients in the α-ketoacid tablet group was much lower than that in the non-α-ketoacid group, indicating the positive role of α-ketoacid in preventing CKD progression (Wang et al., 2019).

In summary, a growing body of evidence confirms that the NF-ƙB signaling pathway plays an important role in renal fibrosis. Inhibitors, including chemical agents, natural products and non-coding RNAs, are available to protect against renal fibrosis progression.

## 6 Conclusion

The common pathway of the progression of all types of AKI and CKD is renal fibrosis; therefore, every attempt to prevent the progression of renal fibrosis could be effective at reducing the burden on the world’s economy. NF-ƙB signaling is considered a pivotal pathway in the progression of renal fibrosis. A growing number of studies suggest that NF-ƙB is a critical mediator of renal fibrosis. In particular, hyperactivation of the NF-ƙB signaling pathway contributes to renal fibrosis. Therefore, inhibiting NF-ƙB signaling may be a prospective approach for the treatment of renal fibrosis. We described a number of lines of evidence indicating that NF-ƙB signaling is a target of renal fibrosis in AKI and CKD as well as in the AKI-to-CKD transition. Unresolved renal inflammation can drive progressive fibrosis. This means that we will face greater challenges in the future to identify more powerful treatments for renal fibrosis. Moreover, natural products are uniquely suited to suppress renal fibrosis, and many isolated compounds have been shown to inhibit the NF-ƙB signaling pathway. This Review covers a variety of strategies for treating renal fibrosis by inhibiting NF-ƙB signaling. Considering the essential role of NF-ƙB in the development of renal fibrosis, the development of therapeutic medicines that inhibit abnormal NF-ƙB signaling is critical for identifying effective therapeutic approaches for treating patients with kidney fibrosis. Future research should focus on the development of new NF-ƙB inhibitors that could prevent and treat renal fibrosis. It is worth noting that the majority of these studies were based on animal and cell experiments. Further research should be conducted to investigate the underlying mechanisms involved to confirm the safety and effectiveness of these treatments in the clinic. Existing studies have focused primarily on inflammation and oxidative stress, as well as the TGF-β-Smad and NF-κB signaling pathways. However, there are limitations, including a lack of diversity in treatment targets. Additional research is needed to explore other cellular and molecular pathways that may be involved.

## Figures and Tables

**Table:** 

**AGEs**	advanced glycation end-products
**AKI**	acute kidney injury
**ATN**	acute tubular necrosis
**CAD**	chronic renal allograft dysfunction
**CKD**, **COX-2**	chronic kidney disease, cyclooxygenase-2
**CTGF**	connective tissue growth factor
**DN**	diabetic nephropathy
**ESRD**	end-stage renal disease
**ECM**	extracellular matrix
**FN**	fibronectin
**HO-1**	heme oxygense-1
**HGMCs**	human glomerular mesangial cells
**HRGECs**	human renal glomerular endothelial cells
**IL-1**	interleukin-1
**IL-6**	interleukin-6
**IKK**	inhibitor of kappa B kinases
**IƙB**	inhibitor of kappa B
**IRI**	ischemia-reperfusion injury
**IgAN**	immunoglobulin A nephropathy
**LncRNAs**	long non-coding RNAs
**LPS**	lipopolysaccharide
**MCP-1**	monocyte chemoattractant protein-1
**MCs**	mesangial cells
**MMT**	macrophage-to-myofibroblast transition
**MN**	membranous nephropathy
**MiRNAs**	microRNAs
**NF-ƙB**	nuclear factor kappa B
**Nrf2**	nuclear factor erythroid-derived 2-related factor 2
**NQO1**	nicotinamide adenine dinucleotide phosphate quinone dehydrogenase 1
**ROS**	reactive oxygen species
**PCAF**	p300/CBP-associate factor
**TCR4**	toll-like receptor 4
**TGF-β**	transforming growth factor-β
**TNC**	tenascin-C
**TNF-α**	tumor necrosis factor-alpha
**TPH-1**	tryptophan hydroxylase-1
**UUO**	unilateral ureteral obstruction
**ZAS**	zymosan activation serum
**5-MTP**	5-methoxytryptophan
